# Increasing donor kidney age significantly aggravates the negative effect of pretransplant donor-specific anti-HLA antibodies on kidney graft survival

**DOI:** 10.3389/fimmu.2025.1574324

**Published:** 2025-04-16

**Authors:** Michiel G. H. Betjes, Judith A. Kal-van Gestel, Dave Roelen, Marcia M. L. Kho, Sebastian Heidt, Annelies E. de Weerd, Jacqueline van de Wetering

**Affiliations:** ^1^ Department of Internal Medicine, Erasmus Medical Center Transplant Institute, University Medical Center, Rotterdam, Netherlands; ^2^ Department of Immunology, Leiden University Medical Center, Leiden, Netherlands

**Keywords:** age donor kidney, antibody-mediated rejection, donor-specific anti-HLA antibodies, graft survival, kidney transplantation

## Abstract

**Background and hypothesis:**

The presence of donor-specific anti-HLA antibodies before kidney transplantation (preDSAs) is associated with decreased graft survival. The hypothesis that increasing donor kidney age is negatively associated with the impact of preDSA on graft survival was investigated.

**Methods:**

Outcome of kidney transplantation in a single center cohort of 2,024 patients transplanted between 2010 and 2020 with a follow-up of at least 3 years was analyzed to assess this relation.

**Results:**

DSAs before transplantation were present in 14% of recipients and showed an independent association with graft loss. The preDSA against HLA class I (2%) or class II (7%) had an adjusted hazard ratio (HR) for death censored graft failure of 5.8 (95% CI 4.4–7.7), while the combination (5%) had an HR of 18.6 (95% CI 13.8–25.1). The preDSA-associated increase in graft failure was caused primarily by an increase in the incidence of antibody-mediated rejection (ABMR), intragraft thrombosis, and primary non-function. These effects were observed more frequently in the deceased donor kidney transplantations compared to living donor kidney transplantations. The incidence of ABMR was not associated with donor kidney age. However, increasing donor kidney age significantly aggravated the negative effect of preDSA on graft survival. For instance, recipients aged ≥65 years transplanted with a deceased donor kidney aged ≥65 years had an uncensored 1- and 3-year graft survival of 83% and 67%, respectively, if transplanted without DSA. This decreased to 56% and 35% if transplanted in the presence of DSA. For comparison, recipients aged ≥65 years of a deceased donor kidney aged <65 years had an uncensored 1- and 3-year graft survival of 92% and 78%, respectively, without preDSA, and if transplanted with preDSA, this decreased to 77% and 69%, respectively.

**Conclusions:**

The negative effect of circulating DSA at the time of transplantation on both early and late death-censored graft survival is heavily influenced by donor age.

## 1 Introduction

The presence of donor-specific anti-HLA antibodies (preDSAs) before kidney transplantation is associated with an increased risk of antibody-mediated rejection (ABMR) and graft loss (1–3). In two large multicenter studies, the effect of DSAs, measured by Luminex single antigen bead assay, was approximately a 10% difference in a 10-year graft survival (4, 5). In the multicenter Dutch PROCARE study, an increased deleterious effect was observed when both DSAs against HLA class I and class II were present (5). Importantly, in these studies, the level of antibodies, which is semi-quantitively expressed as the mean fluorescence intensity (MFI) of the bead signal, was not a discriminating factor for an increased risk of graft loss, and there seems to be no “safe” MFI threshold for preDSAs (4–6). However, the impact of preDSAs on kidney graft survival differs substantially between studies and within different periods of transplantation (7, 8). This may, in part, be explained by DSA-related factors such as complement binding properties and subtype of IgG (9). In addition, donor-related factors, like HLA expression on the allograft (10, 11), an aged immune system in the elderly recipient (12, 13), Fc-receptor genotype (14), and type of immune suppressive regimen, are variable factors, which may determine the incidence of ABMR.

Another contributing factor could be the age of the donor kidney, which has increased substantially over time in Dutch transplant centers. In particular, the European Senior Program (ESP) of Eurotransplant preferentially allocates kidneys from donors aged 65 years and older to recipients of 65 years and older. The results of the latter program in the Netherlands showed that 1- and 3-year graft survival become increasingly poor as the donor age increases (15). Also, donor kidneys aged above 60 years are particularly vulnerable for graft failure due to a variety of causes such as hypovolemic shock, sepsis, and nephrotoxic medication like calcineurin inhibitors (16, 17).

For the recipient, early graft loss in the first years after transplantation has detrimental effects both in terms of increased mortality and a low likelihood of retransplantation (18–20). The impact of preDSAs on graft survival in relation to donor kidney age has not been documented in detail. We hypothesized that older donor kidneys are more vulnerable for immune activation and endothelial inflammation, which can be triggered by circulating preDSAs. Such an effect could lead to increased graft loss with subsequent important clinical consequences, in particular for the elderly recipients.

In this study, we investigated the effect of preDSA on early and late graft failure with a focus on the interaction with donor kidney age using data from a large single-center cohort with a uniform immune suppressive medication regimen in a recent era.

## 2 Patients and methods

This study included all 2,124 consecutive kidney transplantations performed between January 2010 and December 2020 at the Erasmus Medical Center in the Netherlands. The last follow-up date for data analysis was June 2024. Recipients were seen at least once a year in our out-patient clinic, and clinical data were registered in a national database (Netherlands Organ Transplant Registry). All transplantation across the ABO blood group barrier (n = 88) or a current positive complement-dependent cytotoxicity cross-match (n = 12) were excluded from analysis.

Induction therapy was basiliximab in the vast majority of patients. The standard immune suppressive medication protocol was based on tacrolimus (aiming for predose concentrations of 10–15 ng/ml in weeks 1–2, 8–12 ng/ml in weeks 3–4, and 5–10 ng/ml, thereafter) combined with mycophenolate mofetil (starting dose of 1 g b.i.d., aiming for predose concentrations of 1.5–3.0 mg/l) and glucocorticoids. All patients received 50 mg of prednisolone b.i.d. intravenously on days 0–3. Thereafter, 20 mg of oral prednisolone was started and subsequently tapered to 5 mg at month 3 and thereafter stopped within 3 months.

The clinical and research activities being reported are consistent with the Principles of the Declaration of Istanbul as outlined in the “Declaration of Istanbul on Organ Trafficking and Transplant Tourism” and in accordance with the Declaration of Helsinki. All patients gave written informed consent for participating in the Netherlands Organ Transplant Registry database. Approval for assessing additional clinical information was obtained by the institutional review board of the Erasmus Medical Center (MEC-2021-0357 and MEC-2024-0193). Study design and analysis was done in accordance with the *STROBE* statement.

All renal biopsies were *for cause* and were performed in case of progressive loss of graft function. The initial biopsy reviews were rescored following the 2018 Banff Reference Guide (21). ABMR was treated with pulse methylprednisolone and intravenous immunoglobulins (1–2 g/kg bodyweight) with additional plasmapheresis in acute ABMR. Alemtuzumab was administered as second-line treatment in a small number of patients (22).

### 2.1 Outcomes and variables

For data analysis, the outcome of kidney biopsy was further categorized as previously published (23): rejection, recurrence of primary kidney disease, diagnosis of *de novo* kidney disease, and interstitial fibrosis with tubulus atrophy (IFTA). In case of graft failure, the diagnosis of *for cause* kidney biopsies was used to categorize the type of graft failure if no other clinical event could explain the loss of kidney function.

The other graft loss categories were a clinical event leading to irreversible graft failure (e.g., circulatory shock, pyelonephritis, graft thrombosis) and “unknown” if no biopsy was performed and a clinical diagnosis for allograft failure could not be made. Primary non-function is the category of grafts that never had function after transplantation with no other diagnosis than acute tubular necrosis (ATN) as shown by kidney biopsy.

### 2.2 Identification of anti-HLA donor-specific antibodies

Anti-HLA donor-specific antibodies were measured in this study as previously reported (6). A, B, C, DRB1, DRB3, DRB4, DRB5, DQA, DQB, DPA, and DPB were considered for DSA testing. If needed (e.g., in case of anti-DP antibodies), retyping of recipients was performed. During the study period, the methods to detect HLA antibodies have changed from ELISA screening to Luminex screening and subsequently Luminex Single Antigen Bead (SAB) testing. In case of a positive screening, this was followed by antibody identification by SAB assay of either Lifecodes or OneLambda. For the Lifecodes SAB test, data were analyzed using MatchIt. Antibody software version 1.3.1 (Immucor) and results were shown as mean fluorescence intensity (MFI) values, background corrected. Cut-offs were bead specific in combination with a raw MFI of more than 750. For OneLambda, data were analyzed using HLA FUSION antibody software version 3.4.18 (One Lambda) using an MFI of 750 as a cut-off. The percentage of panel reactive antibodies (PRA) at time of transplantation, as determined by complement-dependent cytotoxicity assay, was considered positive when >5%.

### 2.3 Statistical analysis

Differences in patient, donor, and transplant characteristics were assessed by the Fisher’s exact test for categorical variables and Mann–Whitney U test for continuous variables. All p-values were two-tailed. Death-censored graft survival was initially assessed by Kaplan–Meier survival analysis with log-rank statistics for difference between strata of pretransplant DSA. Donor age was categorized as <45 years (n = 487), 45–54 years (n = 464), 55–64 years (n = 595), and 65 years or older (n = 478). Univariate Cox proportional hazards analysis was used to identify clinical and demographic variables associated with rejection and graft survival. Variables considered for analysis were as follows: donor kidney age, age of recipient, re-transplantation, pre-emptive transplantation, positive PRA, pretransplant DSA, type of DSA (anti-HLA class I and/or II), number of HLA mismatches, type of kidney donor (LD, DCD, DBD), male/female, and cold ischemia time. Variables with a p-value of <0.1 in a univariate analysis were subsequently used in the multivariate Cox proportional hazard analysis with stepwise forward regression to calculate adjusted hazard ratios for the outcome (e.g., graft failure) within the categories of pre-DSA compared to no preDSA. For analysis of the incidence of ABMR, the cases with ABMR only and the cases with ABMR mixed with cellular rejection were combined. Interaction terms that met statistical significance (p < 0.05) were included in the multivariate model. All adjusted hazard ratio’s as shown in the results were calculated with the multivariate Cox proportional hazard analysis. Statistical analysis was performed with the software IBM SPSS statistics 21. Statistical significance was met if the p-value was <0.05.

## 3 Results

### 3.1 Baseline characteristics

Baseline characteristics are shown in **Table 1**. The average age of recipients was 56 years, and the average age of donors was 53 years (for LDK 52 years, DBD 56 years, and DCD 56 years), in line with the general trend of increasing numbers of elderly patients receiving a kidney transplant. Over 96% of the recipients were treated with anti-CD25 antibody induction therapy, and 99% continued with the standard protocol of triple immune suppression with tacrolimus as calcineurin inhibitor of choice. The majority of recipients (58%) received a kidney from a living donor, and pre-emptive transplantation was performed in a third of cases. Pretransplant DSAs were present in 14% of transplantations with the majority belonging to anti-HLA class II antibodies, with or without anti-HLA class I antibodies. Of note, the frequency of transplantations with preDSAs decreased from an average of 20% in 2010 to 2014 to 10% in the period 2016–2020. In particular, preDSAs against both classes I and II decreased over the years from an average of 7% to <1% after 2019. The frequency of re-transplantation (14%) was relatively low within this cohort, as was immunization for HLA (positive PRA in 9.5% of cases).

**Table 1:** Kidney transplant recipients and donor kidney clinical characteristics (n = 2,024).

Mean age recipient in years (SD)	55.7 (14.1)
Mean age donor in years (SD)	53.5 (14.5)
Recipient male/female ratio	62/38%
Follow-up in years, median (IQR)	6.2 (4.0–8.3)
Deceased/living donor kidney -DBD type* -DCD type* -Delayed graft function in DBD/DCD -Never functioning graft** Pre-emptive transplantation	42/58% 17% 25% 30%/57% 2% 32%
Cold ischemia time in hours	6.0 ± 5.9
Re-transplantation	16%
PRA*** >5%	9.5%
HLA mismatches (median) Class I Class II Classes I and II	2 1 3
DSA (% positive before transplantation) % within each donor group: LDK/DBD/DCD* HLA class I only HLA class II only HLA class I and II	14% 15%/16%/11% 2% 7% 5%
MFI DSA class I (median and IQR)	4,855 (3,016–8,126)
MFI DSA class II (median and IQR)	2,993 (1,830–5,212)
Induction therapy	96%
Anti-IL-2 receptor antibody	94%
T-cell-depleting antibody	2%
Initial maintenance immune suppression	
Steroids	100%
Tacrolimus/ciclosporin	99%/1%
MMF/azathioprine	99%/1%
Other	<1%

*LDK, living donor kidney; DBD, deceased by brain death; DCD, deceased by circulatory death.

**The category “never functioning graft” includes all kidney transplants that have never functioned sufficiently to allow stopping dialysis.

***PRA, panel reactive antibodies; above 5% indicates the presence of serum cytotoxic anti-HLA antibodies.

### 3.2 Pretransplant DSAs and graft survival

**Figure 1:**
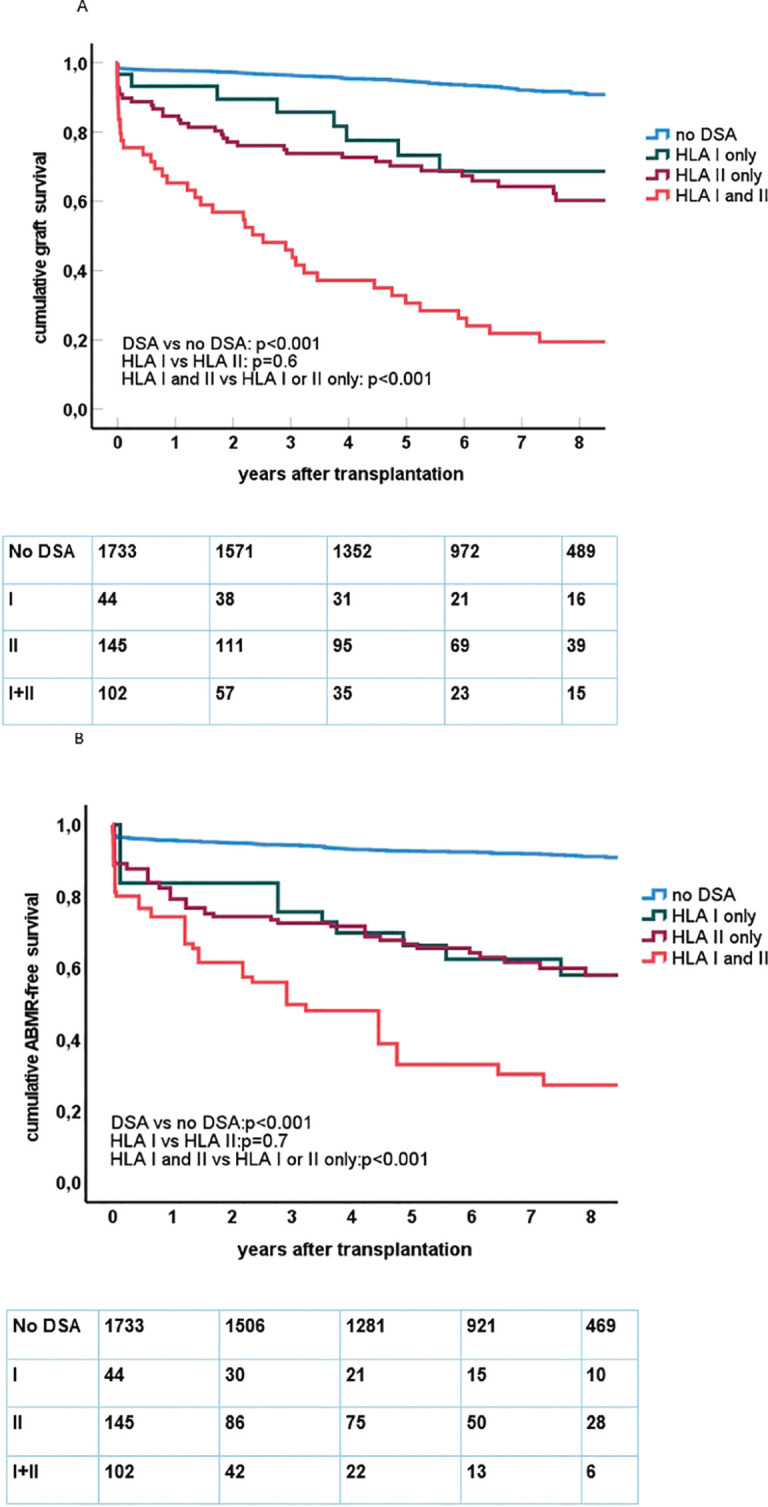
**(A)** Kaplan–Meier curves for death-censored graft survival after kidney transplantation in relation to the presence and type of pretransplant DSAs. Below the figure, the number of at risk at follow-up is shown within each category. Log rank test for comparing different groups of recipients is shown within the figure. **(B)** Kaplan–Meier curves for antibody-mediated rejection (ABMR)-free survival after kidney transplantation in relation to the presence and type of pretransplant DSAs. Below the figure, the number of at risk at follow-up is shown within each category. Log rank test for comparing different groups of recipients is shown within the figure.

The presence of preDSAs was highly associated with decreased graft survival and ABMR-free survival (**Figures 1A, B**). The effect of preDSAs of either class I or II was similar, but the combination of preDSA classes I and II had the largest effect. In particular, early graft loss (**Figure 1A**) and ABMR (**Tables 2**, **3**) shortly after transplantation were observed in the preDSA groups. Multivariable Cox proportional hazard analysis showed an HR for graft failure of 5.8 (95% CI 4.4–7.7) for preDSA class I or II, while the combination had an HR of 18.6 (95% CI 13.8–25.1). Similar results were obtained for the incidence of ABMR.

**Table 2:** Graft loss of deceased donor kidney transplantations (n = 832) at 1 and 3 years after transplantation stratified for the presence of DSAs at time of transplantation in preDSA negative (n = 720) or preDSA positive (n = 112).

	1 year			3 years		
	preDSAneg	preDSApos	p-Value	preDSAneg	preDSApos	p-Value
Graft survival	90.5%	65.2%	<0.001	79.2%	50.0%	<0.001
Death with functioning graft	43 (5.9%)	4 (3.6%)	0.7	100 (13.9%)	5 (4.5%)	0.003
Number of graft loss other than death	33 (4.6%)	35 (31.2%)	<0.001	50 (6.9%)	51 (45.5%)	<0.001
Cause of graft loss						
All biopsy-proven rejections	15 (2.1%)	21 (24.1%)	<0.001	21 (2.9%)	31 (27.6%)	<0.001
TCMR*	8 (1.1%)	7 (6.2%)	0.001	12 (1.7%)	10 (8.9%)	<0.001
ABMR*	5 (0.7%)	8 (7.1%)	<0.001	6 (0.8%)	12 (10.7%)	<0.001
Mixed-type rejection	2 (0.3%)	6 (5.3%)	<0.001	3 (0.4%)	9 (8.0%)	<0.001
Interstitial fibrosis/tubulus atrophy	0	1 (0.9%)	0.9	2 (0.3%)	2 (0.9%)	0.8
Recurrence of primary kidney disease	0	0	–	2 (0.3%)	1 (0.9%)	0.7
Kidney injury/disease**	1 (0.1%)	1 (0.9%)	0.6	5 (0.7%)	3 (2.7%)	0.6
Renal artery/vein thrombosis or intragraft thrombi	6 (0.8%)	4 (3.6%)	0.03	6 (0.8%)	4 (3.6%)	0.03
Unknown	1 (0.1%)	0	0.9	3 (0.4%)	1 (0.9%)	0.7
Primary non-function	8 (1.1%)	8 (7.1%)	<0.001	8 (1.1%)	8 (7.1%)	<0.001
Other	2 (0.3%)	0	0.9	3 (0.4%)	1 (0.9%)	0.8

All data are given in number of cases, and the % of the total number of transplantations within the stratum is in parenthesis.

*TCMR, T-cell-mediated rejection; ABMR, antibody-mediated rejection.

**Kidney injury/disease is the category including events or diseases causing irreversible kidney injury leading to graft loss. The category “unknown” indicates that no kidney biopsy was performed, and no clinical cause of graft loss was established. ns, not significant (p > 0.05).

**Table 3:** Graft loss of living donor kidney transplantations (n = 1,192) at 1 and 3 years after transplantation stratified for the presence of DSA at time of transplantation in preDSA negative (n = 1,011) or preDSA positive (n = 181).

	1 year			3 years		
	preDSAneg	preDSApos	p-Value	preDSAneg	preDSApos	p-Value
Graft survival	96.0%	89.1%	<0.001	91.9%	72.4%	<0.001
Death with functioning graft	25 (2.4%)	3 (1.6%)	ns	59 (5.8%)	8 (4.4%)	ns
Number of graft loss other than death	12 (1.2%)	17 (9.3%)	<0.001	23 (2.3%)	42 (23.2%)	<0.001
Cause of graft loss						
All rejections	4 (0.4%)	9 (4.9%)	<0.001	12 (1.2%)	28 (15.5%)	<0.001
TCMR*	2 (0.2%)	3 (1.6%)	0.02	7 (0.7%)	10 (5.5%)	<0.001
ABMR*	1 (0.1%)	1 (0.5%)	ns	3 (0.3%)	6 (3.3%)	<0.001
Mixed-type rejection	1 (0.1%)	6 (3.3%)	<0.001	2 (0.2%)	12 (6.6%)	<0.001
Interstitial fibrosis/tubulus atrophy	0	0	–	2 (0.2%)	0	ns
Recurrence of original disease	0	0	–	1 (0.1%)	1 (0.5%)	ns
Kidney injury/disease**	3 (0.3%)	0	ns	3 (0.3%)	1 (0.5%)	ns
Renal artery/vein thrombosis or intragraft thrombi	1 (0.1%)	5 (2.7%)	<0.001	1 (0.1%)	5 (2.7%)	<0.001
Unknown	0	0	–	0	2 (1.1%)	0.02
Primary non-function	1 (0.1%)	1 (0.5%)	ns	1 (0.1%)	1 (0.5%)	ns
Other	3 (0.3%)	2 (1.1%)	ns	3 (0.3%)	4 (2.2%)	0.01

All data are given in number of cases, and the % of the total number of transplantations within the stratum is in parenthesis. *TCMR, T-cell-mediated rejection; ABMR, antibody-mediated rejection.

**Kidney injury/disease is the category including events or diseases causing irreversible kidney injury leading to graft loss. The category “unknown” indicates that no kidney biopsy was performed, and no clinical cause of graft loss was established. ns, not significant (p > 0.05).

Data on graft survival and cause of graft loss was performed separately for living (LD) and deceased donor (DD) kidney transplantations because of significant differences in effect size of preDSA (**Tables 2**, **3**). At 1 year after transplantation, the graft survival in the preDSA-positive DD versus the preDSA-negative DD group was significantly lower (65.2% vs. 89.5%). This was mainly caused by an increased incidence of AMBR (whether or not “mixed” with TCMR) from 1% to 12.4%, primary non-function (1.1% vs. 7.1%), and thrombosis (0.8% vs. 3.6%), usually of the renal vein or artery. At 3 years post-transplantation, the uncensored graft survival in the preDSA DD group was 50% (79% in the DD group without preDSA), largely attributable to a further increase in ABMR-related graft loss to 18.7%. Within the LD group, similar negative effects, although to lesser extent than within the DD group, were seen in the preDSA-positive group. At 1-year, graft loss other than death was increased (9.3% vs. 1.2%). Specifically, ABMR-related graft loss (3.8% vs. 0.2%) and kidney thrombosis were increased (2.7% vs. 0.1%). At year 3, the cumulative incidence of graft loss other than death was 10 times higher in the preDSA group (23.2 vs. 2.3%).

### 3.3 The negative impact of preDSAs on graft survival is largely determined by older age of the donor kidney

**Figure 2:**
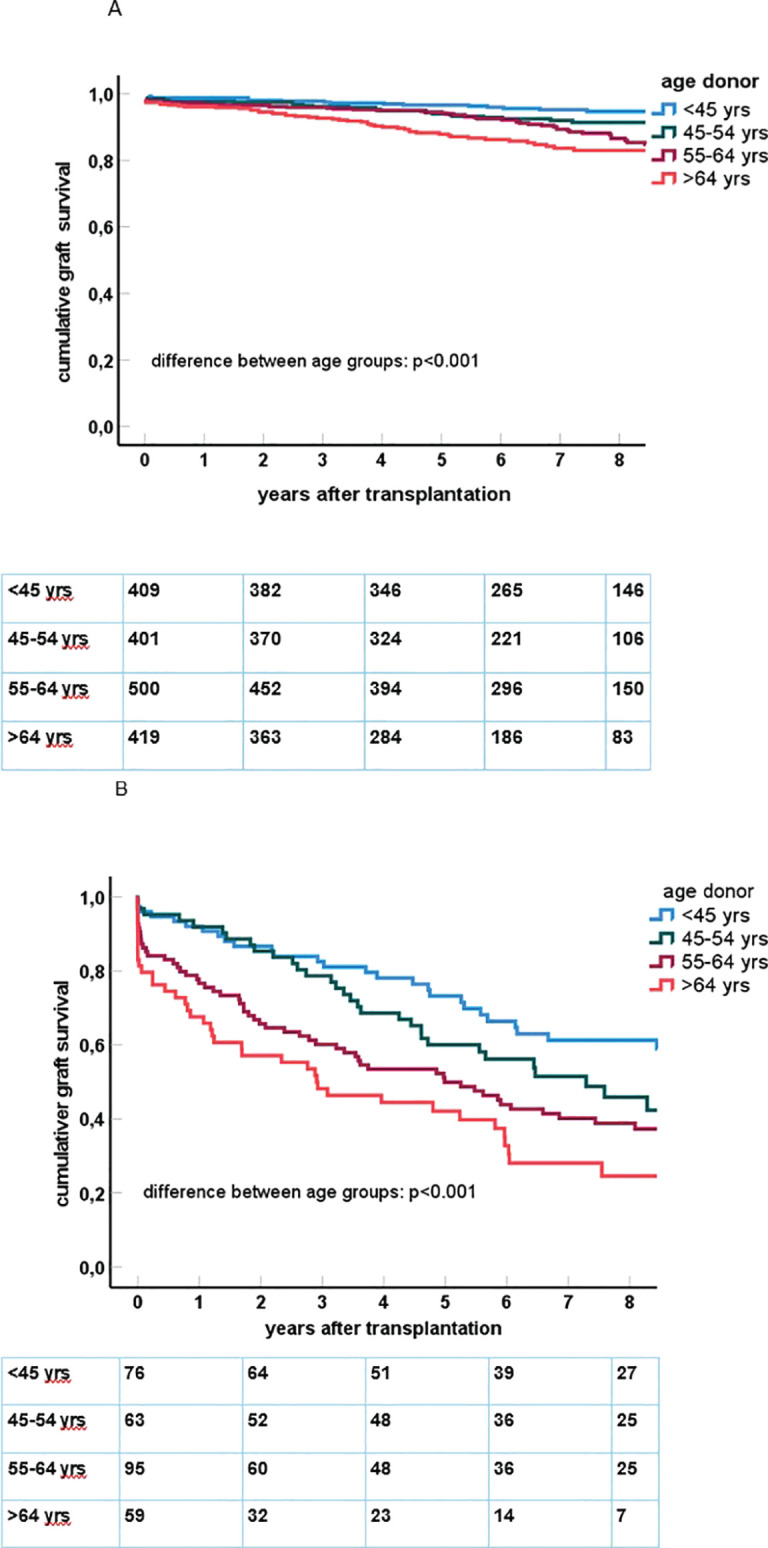
**(A)** In the upper figure, Kaplan–Meier curves for death-censored graft survival after kidney transplantation in the group of recipients of a donor kidney without pretransplant DSA are shown. The number of cases within each age category is n = 409 (age <45 years), n = 401 (45–54 years), n = 500 (55–64 years), n = 419 (65 years and older). **(B)** In the lower figure, Kaplan–Meier curves for death-censored graft survival after kidney transplantation in the group of recipients of a donor kidney with pretransplant DSA are shown. The number of cases within each age category is n = 76 (age <45 years), n = 63 (45–54 years), n = 95 (55–64 years), n = 59 (65 years and older).

The age category of the donor kidney was significantly associated with the impact of preDSA on graft survival (**Figure 2**). Multivariate Cox proportional hazard analysis was performed to assess the effect of preDSA in different age groups of donor kidneys (**Table 4**; **Supplementary Table 1** for all significant variables). Within every age group, the hazard ratio of preDSAs against HLA class I or II only or the combination thereof was significantly associated with an increase in the hazard ratio for graft loss. The hazard ratio of preDSA was roughly similar in all donor age categories, but this resulted in a much higher absolute % of graft loss in the older donor age categories as can be appreciated from **Figure 2**. Specifically, recipients aged ≥65 years transplanted with a DD kidney within the Eurotransplant Senior Program (n = 213) had an uncensored 1- and 3-year graft survival of 83% and 67%, respectively, without preDSAs, but if transplanted with preDSAs, this decreased to 56% and 35%, respectively. If recipients aged ≥65 years received a DD kidney of <65 years of age (n = 131), the uncensored 1- and 3-year graft survival was 92% and 78%, respectively, without preDSAs, and if transplanted with preDSAs, this decreased to 77% and 69%, respectively. In both LD and DD groups the donor age itself was not associated with an increase in the incidence of ABMR (data not shown). Thus, it is the age and the type (LD or DD) of donor kidney, which are the main determinants of the impact of preDSA on graft survival.

**Table 4:** The hazard ratios for graft failure within different categories of pre-transplant DSA compared to those of recipients without pre-transplant DSA, analyzed per donor age group.

Donor age group	Type of donor-specific anti-HLA antibodies		
	HLA class I	HLA class II	HLA classes I and II
<45 years	HR^*^ 5.8 (1.9–17.5)	HR 7.4 (3.4–16.2)	HR 29.4 (12.9–66.4)
45–54 years	HR 2.7 (0.6–11.2)	HR 4.4 (2.2–9.1)	HR 18.0 (10.2–31.9)
55–64 years	HR 5.5 (2.6–1.6)	HR 5.4 (3.4–8.8)	HR 13.1 (8.3–20.8)
65 years and older	HR 6.5 (2.4–18.2)	HR 5.3 (3.0–9.1)	HR 15.9 (9.3–27.2)

^*^Hazard ratio (HR) with 95% confidence interval between parenthesis. Within the defined donor age group, a multivariate Cox proportional hazard analysis was performed including the clinically relevant variables that had a p < 0.1 in the univariate Cox proportional hazard analysis. The categories of pre-transplant DSA were defined as anti-HLA class I only, anti-HLA class II only, or the presence of both anti-HLA classes I and II before transplantation.

## 4 Discussion

The current study shows that transplantation with a negative CDC-XM but in the presence of DSAs is associated with increased early and late graft loss. This is primarily because of ABMR, but an increased risk for primary non-function (in case of DD kidneys) and thrombosis was also observed. In particular, the combination of preDSAs against both HLA classes I and II carried a high risk for graft loss. These findings regarding the negative effects of preDSA are not consistent in the literature, but they have been observed in three large cohort studies (4, 5, 24) with a separate analysis of cause of graft failure when DSAs are present (2, 6). In the current study, the combination of preDSA against both HLA classes I and II carried the highest risk for graft loss, while preDSAs for either class I or II had a similar lower risk. This finding is in agreement with the results of the Swiss Transplant Cohort Study (24) and of the Dutch National PROCARE study involving 4,724 transplantations performed between 1995 and 2006 (5). After publication of these results, transplantations with this combination of preDSAs dropped sharply in our center. Of note, a German cohort study did not observe such an effect of combined preDSAs for classes I and II on graft survival, which may be related to differences in type of maintenance immune suppressive drugs and the use of desensitization procedures (4).

In our center, basiliximab instead of lymphocyte depleting induction is given. This differs from practices in, for example, the United States, where over 60% of recipients receive T-cell depleting induction therapy (25). As discussed in a previous report (6), approaches to induction therapy vary substantially, and in many European countries, basiliximab is the first-line therapy in the vast majority of recipients. Although the KDIGO guidelines suggest using a lymphocyte-depleting agent for kidney transplant recipients at high immunologic risk, this strategy was not adopted in the past in many (European) centers, as the evidence level of this recommendation was modest, and most data were from deceased donor transplantation. In addition, the negative effects on graft survival of preDSAs was and is a matter of debate, and others reported a general favorable outcome of transplantations with preDSAs using non-depleting antibody induction (26, 27).

Currently, we avoid transplantation across preDSAs as much as possible by the use of an effective computer algorithm-supported national cross-over program in case of a living donor (28, 29) and a desensitization program involving imlifidase for deceased donor kidney transplantations (30).

The age of older kidney donors has increased by almost 10 years in our center compared to that of 20 years ago (23). Furthermore, within the ESP, DD kidneys aged 65 years or older are preferentially allocated to recipients aged 65 years or older, and the number of older recipients has been growing over the years. An analysis of the results of kidney transplantation in the elderly recipients within the Netherlands in the period 2005 to 2015 demonstrated that the ESP resulted in a 1-year graft survival of 77% and a death-censored 1-year graft survival of 87% (15). These results are in accordance with our study, and based on the abovementioned PROCARE study, it is likely that a similar percentage of transplantations within ESP was performed in the presence of preDSAs and thus substantially influenced the results of that study.

It is a consistent finding that the age of the donor kidney is related to the risk for graft failure, especially in deceased donor transplantation (16, 17). This may be multifactorial as older kidneys are more vulnerable for any form of acute kidney injury including cold ischemia time. In addition, data from the ESP showed that the older deceased donor kidneys reached an eGFR <30 ml/min at 1 year in >50% of recipients vs. 26% in recipients of a young donor kidney (15) Thus, the functional reserve capacity of the older donor kidneys is frequently low with the consequence of reaching graft loss at an earlier time point in case of adverse events such as ABMR. Older donor kidneys are known to be more immunogenic and associated with a higher risk for TCMR (23, 31), but in this cohort, we could not find an independent association between the age of the donor kidney and the risk for clinically relevant ABMR. However, as no per protocol biopsies were performed, we cannot rule out the possibility that subclinical ABMR may be more frequently seen in kidneys of older age donors. Nevertheless, preDSAs significantly increased the risk for ABMR, which led to a substantial graft loss within the first year in every donor kidney age group. In particular, the combination of old donor age and deceased donation led to a much-shortened graft survival for reasons mentioned above, but the hazard ratio was in a similar range for the different donor kidney age groups. Transplantation without preDSAs, using a triple immune suppression regime with anti-CD25 induction, shows a death-censored graft loss of <5% at 1 year, even when older DD kidneys were used (of which half were DCD type kidneys). Also, long-term survival is remarkably good in kidney transplantations without preDSAs. Although relatively few transplantations are performed with preDSAs, the negative impact on graft survival is of such magnitude (specifically in the older DD kidney group) that graft survival of the whole group is significantly affected. Comparing results between transplantation centers or over time should therefore always be considered in the context of the frequency of transplantations with preDSAs and donor kidney age.

In kidney transplant programs worldwide, the mean age of recipients is steadily increasing (32); in our center, half of the transplantations are performed in recipients 60 years of age and older with an active approach to list elderly patients for transplantation (16). An aged immune system and frailty can make elderly recipients prone to side effects of immune suppressive drugs and infections, especially after anti-rejection treatment (13, 33). Therefore, the benefits of kidney transplantation need to be carefully balanced against these risks. In addition, early graft loss is a dramatic event in the elderly recipients as it increases mortality after returning to dialysis, and relisting is even more infrequent than for the total population of recipients with early graft loss (18, 19). Instead, if an elderly recipient does not experience early graft loss, the risk of death-censored graft loss beyond 1 year after transplantation is remarkably low (16).

Based on the data presented, preDSAs should be avoided as much as possible, at least in case of an older DD kidney and entirely in case of preDSAs against both HLA classes I and II. Clearly, the results are much better for preDSA and a young LD kidney, but even then, the risk of late graft loss was substantially increased.

The strength of the current study is the large number of kidney transplantations with a uniform immune suppressive regimen based on anti-CD25 induction therapy and maintenance with tacrolimus, MMF, and prednisone with follow-ups for at least 3 years after transplantation. As only *for cause* biopsies were performed, the cumulative incidence of ABMR may have been even higher than reported. However, the cause of graft failure for virtually all patients was documented, which revealed that primary non-function (with no signs of ABMR in the biopsy) and graft thrombosis were much more frequently seen in association with preDSAs. The deleterious effects of preDSAs may be better predicted if a flow cytometry crossmatch assay is positive (34). However, in a recent analysis in our center, we could not confirm a relation between height of MFI or flow cytometry crossmatch-positive DSAs and graft survival (6).

In conclusion, increasing donor kidney age significantly aggravates the negative effect of pretransplant donor-specific anti-HLA antibodies on graft survival after kidney transplantation.

## Data Availability

The original contributions presented in the study are included in the article/**Supplementary Material**. Further inquiries can be directed to the corresponding author.
