# Supplementary material for: Increasing donor kidney age significantly aggravates the negative effect of pretransplant donor-specific anti-HLA antibodies on kidney graft survival
Source: Front Immunol. 2025 Apr 16;16:1574324. doi: 10.3389/fimmu.2025.1574324 (PMC12040950; doi:10.3389/fimmu.2025.1574324)
Supplement: Supplementary file 1 [file DataSheet1.pdf]

Table 1 supplement

The hazard ratio's for graft failure within different categories of pre-transplant DSA as compared to recipients without pre-transplant DSA and other clinical variables , analyzed per donor age group.

Donor age group	significant variables within the multivariate Cox proportional hazard analysis	
	Variables in the equation	Hazard ratio with 95% CI
<45 years	DSA anti-HLA class I only*	5.8 (1.9-17.5)
	DSA anti-HLA class II only*	7.4 (3.4-16.2)
	DSA anti-HLA class I and II*	29.4 (12.9-66
	Age recipient per year older	0.97 (0.95-0.99)
	LD vs DD kidney	0.45 (0.24-0.85)
	no PRA vs PRA positive	0.29 (0.12 -0.70)
45-54 years	DSA anti-HLA class I only	2.7 (0.6-11.2)
	DSA anti-HLA class II only	4.4 (2.2-9.1)
	DSA anti-HLA class I and II	18.0 (10.2-31.9)
	LD vs DD kidney	0.45 (0.27-0.76)
55-64 years	DSA anti-HLA class I only	5.5 (2.6-1.6)
	DSA anti-HLA class II only	5.4 (3.4-8.8)
	DSA anti-HLA class I and II	13.1(8.3-20.8)
	LD vs DD kidney	0.36 (0.25-0.53)
65 years and older	DSA anti-HLA class I only	6.5 (2.4-18.2)
	DSA anti-HLA class II only	5.3 (3.0-9.1)
	DSA anti-HLA class I and II	15.9 (9.3-27.2)
	Age donor kidney per year older	1.05 (1.01-1.10)
	LD vs DD kidney	0.44 (0.29-0.67)

Within the defined donor age group, a multivariate Cox proportional hazard analysis was performed including the clinically relevant variables that had a $p<0.1$ in the univariate Cox proportional hazard analysis. The variables which remained within the equation after stepwise forward conditional analysis are given. *The categories of pre-transplant donor-specific antibodies (DSA) were defined as anti-HLA class I only, anti-HLA class II only, or the presence of both anti-HLA class I and II before transplantation and the hazard ratio's are given compared to no pre-transplant DSA. Living donor kidney (LD); deceased donor kidney (DD);panel reactive antibodies (PRA) are considered positive when >5%

*
